# The Janus-faced nature of complement in hemodialysis: interplay between complement, inflammation, and bioincompatibility unveiling a self-amplifying loop contributing to organ damage

**DOI:** 10.3389/fneph.2024.1455321

**Published:** 2024-12-03

**Authors:** Bernard Canaud, Peter Stenvinkel, Rebecca Scheiwe, Sonja Steppan, Sudhir Bowry, Giuseppe Castellano

**Affiliations:** ^1^ School of Medicine, University of Montpellier, Montpellier, France; ^2^ Dept of Renal Medicine, Karolinska University Hospital, Stockholm, Sweden; ^3^ Fresenius SE & Co. KGaA, Bad Homburg, Germany; ^4^ Dialysis-at-Crossroads (D@X) Advisory, Bad Nauheim, Germany; ^5^ Center for Hemolytic Uremic Syndrome (HUS) Prevention, Control, and Management at the Nephrology and Dialysis Unit, Fondazione Scientific Institute for Research, Hospitalization and Healthcare (IRCCS) Ca’ Granda Ospedale Maggiore Policlinico, Milan, Italy

**Keywords:** inflammation, complement, biocompatibility, dialysis, technology, clinical impact, chronic kidney disease

## Abstract

In hemodialysis (HD), complement activation, bioincompatibility, and inflammation are intricately intertwined. In the 1970s, as HD became a routine therapy, the observation of complement pathway activation and transient leukopenia by cellulosic dialysis membranes triggered the bioincompatibility debate and its clinical relevance. Extensive deliberations have covered definitions, assessment markers, scope, and long-term clinical consequences of membrane-dependent bioincompatibility reactions. While complement pathways’ interplay with coagulation and inflammation has been delineated, HD’s focus has primarily been on developing more biocompatible membranes using advanced technologies. Recent advances and understanding of the current HD delivery mode (4-hour sessions, thrice weekly) suggest that factors beyond membrane characteristics play a significant role, and a more complex, multifactorial picture of bioincompatibility is emerging. Chronic activation of the complement system and persistent low-grade “uremic inflammation” in chronic kidney disease (CKD) and HD lead to premature inflammaging of the kidney, resembling aging in the general population. Cellular senescence, modulated by complement activation and the uremic milieu, contributes to chronic inflammaging. Additionally, the formation of neutrophil extracellular traps (NETs, process of NETosis) during HD and their biological activity in the interdialytic period can lead to dialysis-induced systemic stress. Thus, complement-inflammation manifestations in HD therapies extend beyond traditional membrane-related bioincompatibility consequences. Recent scientific knowledge is reshaping strategies to mitigate detrimental consequences of bioincompatibility, both technologically and in HD therapy delivery modes, to improve dialysis patient outcomes.

## 1 The complement – hemodialysis axis: the genesis of bioincompatibility

The association of complement system pathways with HD therapy dates back to the early days of maintenance dialysis. In the late sixties and early seventies, Craddock et al. reported acute pulmonary dysfunction caused by complement-mediated leukostasis with cellulose-based membranes (1–3), mainly Cuprophan being available at scale to meet the growing HD demand. Their findings essentially lead to awareness of the bioincompatibility topic in HD (4). Two main ramifications emerged. Firstly, a debate ensued regarding the clinical effects of bio*in*compatibility and chronic complement activation (4, 5), leading to extensive research on mechanisms and proposed explanations for clinical sequelae (5, 6). Secondly, complement activation-leukopenia caused by ‘natural’ cellobioses’ high percentage of hydroxyl groups (7) prompted membrane manufacturers to mitigate these effects. Consequently, there was a decline in cellulose-based HD membranes, with a preference shifting towards synthetic polymers, particularly those based on polysulfone-based (8). Continued advancements in HD therapies involve enhanced removal of middle-sized uremic toxins using high-flux membranes and convective therapies, coupled with the utilization of ultrapure dialysis fluids (9).

A significant body of literature details HD membrane-related complement activation and transient leukopenia (10). This narrative assay aims to explore beyond classical measures of bio*in*compatibility, examining the interplay of residual complement-leukopenia, inflammation, oxidative stress, and endothelial dysfunction pathways in light of newer integrated biomarkers of bioincompatibility.

## 2 The classical scientific perspective of bioincompatibility in hemodialysis

Even in the early stages of artificial kidney development, it was evident that blood interacting with extracorporeal circuit (ECC) surfaces would elicit reactions (11). Initially, clotting posed a challenge until optimized heparin anticoagulation regimens allowed for completion of 3-4-hour HD sessions (12). Despite heparin’s effectiveness in preventing clot formation in the ECC, it does not inhibit coagulation-platelet or complement-leukocyte pathway activation (13). Blood-material interaction studies revealed sub-macroscopic coagulation cascade activation, assessed by markers like thrombi-antithrombin III (TAT), d-dimer, or prothrombin fragment_F1+2_ (14), persisting even with heparin use. Complement activation, effectively inhibited only by divalent cation (Ca^++^/Mg^++^) chelating anticoagulants like citrate, continues during HD initiation, reaching peak levels within 15-30 minutes (5). Several authors have detailed the membrane-specific mechanisms and kinetics of activation of complement by different membranes (15–18). Pure cellulose membranes, irrespective of the marker used for assessment (e.g., C3a, C5a or terminal complement complex, TCC), peak at around 15 minutes (19), while modified cellulosic and synthetic polymer membranes reach lower peak levels later, usually between 15 to about 60 minutes (20). Post-peak, complement activation decreases but does not revert to pre-dialysis levels. Transient leukopenia sees neutrophil levels increase beyond pre-dialysis levels by HD session end.

Observations of anaphylatoxin formation (C5a, C3a) based on dialyzer membrane type increased focus on other bio*in*compatibility-related issues in HD (21–23). Although rare, true or pseudo membrane-related hypersensitivity reactions (HSRs) are feared complications (24–26). This is exemplified by AN69 (polyacrylonitrile) membrane-induced anaphylactic shock reactions concurrent with angiotensin-converting enzyme inhibitors (ACEI) treatment (27). In this case, AN69’s negative charge prompts increased bradykinin formation, with ACEIs preventing its degradation in renal failure patients leading to a sudden and brisk release with vasodilation shock (28, 29). Another recent example has surfaced in the form of small, disseminated outbreaks of HSR-like reactions associated with the use of polysulfone membranes (30, 31), prompting questions regarding their role in complement mimicry, resembling complement activation-related pseudo allergy (CARPA) observed with certain chemicals and nanomedicines (23, 32, 33). While the membrane is the centrepiece of HD, offering the largest surface area and serving as a stimulus for blood-material interaction, the entire ECC, with diverse polymeric materials for the potting, tubing, and bubble trap chamber, and conduit configurations, impacts rheological and bioreactive conditions. This includes dialysis fluid residues (i.e., microbial derived products, endotoxins, or components like acetate) enhancing bio*in*compatibility reactions (34).

## 3 An alternative perspective on bioincompatibility in hemodialysis

Beyond HD’s beneficial and life-sustaining detoxification, along with its bio*in*compatibility consequences, a picture of its unphysiological nature and systemic, long-lasting effects on patient wellbeing emerges (35–38). Evidence indicates that repetitive HD contributes to dialysis-induced systemic stress, leading to morbidity by affecting multiple body functions and organs (39–42). Beyond physiochemical membrane properties, other mechanisms related to CKD condition itself, and HD delivery modes induce biological changes with systemic consequences (37, 43, 44).

### 3.1 The Janus-faced role of complement: chronic kidney disease, uremia and hemodialysis procedures

#### 3.1.1 Complement mediates kidney diseases

**Figure 1:**
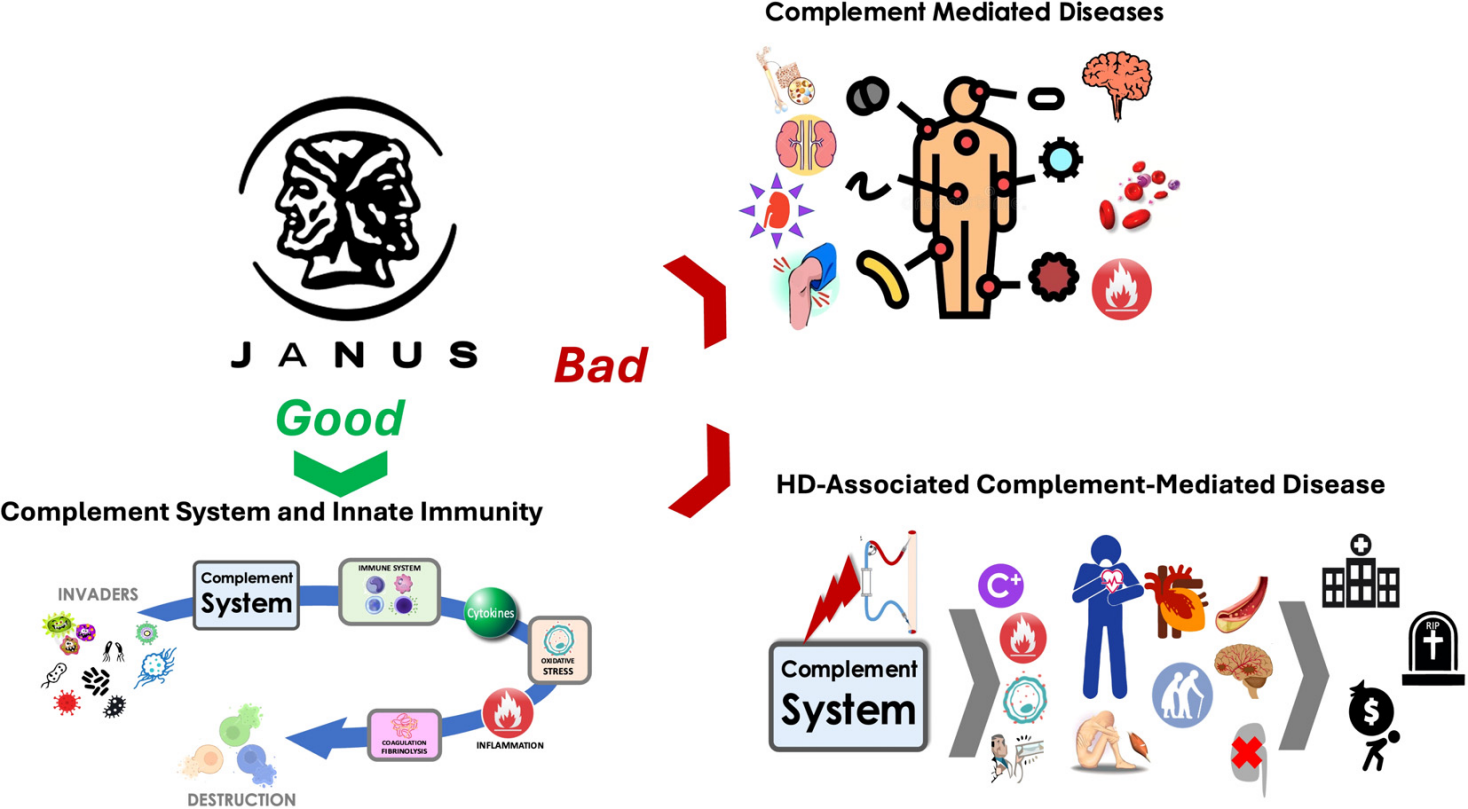
The complement system plays a dual role as depicted here: one side represents its essential role ‘Good’ in innate immunity as a defender against foreign invaders, while the other side reflects its harmful contribution ‘Bad’ to complement-mediated diseases, including those associated with hemodialysis.

While the complement cascade is part of innate immunity against invading pathogens (**Figure 1**), it may also serve as a mediator in various diseases and injuries when imbalance occurs in complement activation and inhibition system (45, 46). Clinical evidence strongly links complement activation to the pathogenesis of several diseases, including renal diseases, contributing for example to the progressive replacement of functioning nephrons by fibrosis (47, 48). Liver-produced circulating complement components activated through classical, mannose-binding lectin, or alternative pathways, mediate pathologic processes (48). Autoantibody-initiated forms of glomerulonephritis (lupus nephritis, anti-glomerular basement membrane disease), anti-neutrophil cytoplasmic autoantibody-induced or membranoproliferative glomerulonephritis, atypical forms of hemolytic uremic syndrome (aHUS), membranous nephropathy (MN), C3 glomerulopathy (C3G), ischemic-reperfusion injury of transplanted kidney, and antibody-mediated renal allograft rejection occur when the immune system becomes overly active (45, 48, 49). It is noteworthy that the kidney was one of the first organs identified as a target of complement-mediated inflammation. The alternative complement pathway is activated in early-stage CKD, contributing to its progression.

Systemic complement activation, particularly fragments Ba and C5b-9, correlates with vascular dysfunction in stage III/IV CKD patients (33, 50). Overactivation of the alternative complement pathway in renal disease, coupled with persistent low-grade inflammation and oxidative stress in early CKD stages, increases sensitivity to complement reactivity with ECC components in HD therapy (51, 52). Complement-mediated kidney diseases result in debilitating symptoms, significantly affecting patients’ quality of life, particularly when permanent HD is required (46, 47, 51).

#### 3.1.2 Key complement factors and cytokines defining uremia

As chronic kidney disease advances, particularly in advanced stages, both complement and inflammation pathways experience increasing activation, reaching peak intensity by CKD stage 5 (53). On one hand, it is now well recognized that repetitive low-grade complement activation induced by hemodialysis, despite the use of synthetic polymer membranes, is associated with higher mortality, particularly of cardiac origin (17, 18, 54). On the other hand, it is also well recognized that inflammation, oxidative stress, and complement activation are interlinked and mutually reinforce their deleterious effects. In end-stage kidney disease (ESKD), elevated plasma levels of complement factor D, Ba, and cytokines such as interleukins: IL-1ß, IL-6, IL-8, IL-10, IL-18 and TNF-alpha as well as leptin, resistin and visfatin) define the uremic syndrome and contribute to the pro-inflammatory status of uremia (55–57), (58). However, establishing the biological reactivity or toxicity of these substances has been challenging, as not all compounds at elevated levels express sufficient toxicity to merit their removal in HD (59). Scientific or clinical evidence regarding the toxic potential of individual compounds is often insufficient for classifying them as uremic toxins (60). A comprehensive review categorized substances based on overall experimental and clinical evidence, as well as the number of biological systems most frequently affected, such as inflammatory, cardiovascular, or fibrogenic systems, considered major players in the high morbidity and mortality in CKD (61). All the complement and inflammation markers listed above fall in the uremic toxins category, with IL-6, TNF-alpha and IL-1ß, IL-8 being the uremic toxins with the highest toxicity score (62).

#### 3.1.3 Hemodialysis’ dual role: correcting and fuelling activation of complement and inflammation

Whether generated by CKD or the subsequent condition of uremia, the elevated concentrations of complement-inflammation uremic toxins described above must be effectively reduced by HD.

Unlike most small uremic toxins (< 500 Da), markers of complement and inflammation classified as uremic toxins are considerably larger, requiring the use of membranes with higher ‘flux’ for their elimination (63). The flux of a given uremic solute through a dialysis membrane reflects mean pore size, sieving capabilities for molecules of a certain size (molecular weight) and operating conditions (blood and dialysate flow, transmembrane pressure, surface area, treatment time) that drive solute flux. It is important to select a membrane that is not overly permeable to prevent the leakage of essential nutrients and compounds, including albumin (64). HD, based on size-exclusion principles, is a compromise between efficiently removing unwanted (‘toxic’) compounds and restricting essential plasma component elimination (59). The application of convective treatment modalities, such as online-hemodiafiltration (OL-HDF), enhances the efficiency of removing markers of both complement and inflammation compared to ‘standard high-flux’ HD (65, 66, 67). This property may corroborate findings from a recent independent large-scale trial showed that high-volume OL-HDF (HV-HDF) reduces patient mortality by 23% relative to standard dialysis treatment (68).

In hemodialysis (HD), restoring the proper balance between removing or preventing the generation of detrimental complement and inflammatory compounds is crucial, as their generation inevitably occurs during the procedure. Complement, predominantly generated by membrane material activation pathways, and pro-inflammatory endotoxins, potentially arising in dialysis fluids due to bacterial growth in water supply systems (69), require strategies for their reduction. These include using dialysis membranes with high biocompatibility and endotoxin-retention capabilities (e.g., the recently developed CorAL dialyzers with advanced hydrogel technology) and employing ultrapure water prepared by reverse osmosis passing through special endotoxin adsorbing filters (20, 70, 71).

#### 3.1.4 The mode of dialysis delivery impacts inflammation and complement

Hemodialysis therapy is commonly administered thrice-weekly in 4-hour sessions, balancing clinical targets and limited financial resources for treating an increasing number of ESKD patients. Intermittent HD regimens expose patients to continuous hemodynamic stress and persistent low-grade inflammation (9, 37), causing acute stress by rapid fluid depletion during dialysis sessions, and chronic stress of extracellular fluid accumulation during interdialytic periods (9). Intensified hemodialysis, based on longer treatment times or more frequently performed home-based therapies, result in lower levels of inflammation markers, myocardial cellular damage, and congestion, improving survival of chronic HD patients compared with conventional HD (41). Cyclic hemodynamic perturbances result in perfusion-dependent, gradually increasing injury and systemic inflammation affecting various vascular beds, especially in the heart, gut, brain, and potentially the kidney (36, 72).

The choice of treatment modality also influences the activation of the complement-inflammation axis. OL-HDF is associated with reduced inflammation and improved survival (73, 74). Comparing OL-HDF to high-flux HD, Ramirez et al. showed that high convective transport (OL-HDF) decreases microinflammation by attenuating endothelial dysfunction through modulating proinflammatory cells or a complex interaction involving the removal of a wider range of uremic toxins^71^. Strategies limiting endothelial damage during dialytic therapies, aiming to curtail inflammation, are considered essential for improving cardiovascular outcomes (cardioprotection) in the dialysis population (38, 75).

Not all substances retained in blood in ESKD have been shown to contribute to the uremic syndrome, possibly due to insufficient scientific and clinical evidence demonstrating their toxicity (61). Complement factor D a large (24,000 Da) molecule, as well as free light chain immunoglobulin components (76) (K and L) or alpha 1 Microglobulin expressing toxicity (77), are candidates for elimination during dialysis. A prospective clinical trial comparing OL-HDF with HF-HD showed a significant decrease in pretreatment serum concentrations of complement factor D in OL-HDF-treated patients, emphasizing treatment modality-related alleviation of complement-inflammation factors (78).

### 3.2 Chronic consequences of complement activation: cellular senescence and inflammaging

We have discussed the mechanisms and role of HD-related complement activation, contributing to (relatively) short-term effects of inducing inflammation, promoting coagulation, and mediating various kidney diseases and cardiovascular events. Complement, as part of the innate immune system, has three overarching physiological functions: defending against pyrogenic bacterial infection, bridging adaptive immunity, and disposing of immune complexes and products of inflammatory injury (48). Persistent, low-grade “uremic inflammation,” associated with increased pro-inflammatory cytokines, resembles the process termed “inflammaging” which is observed in various chronic diseases and aging (79, 80).

The aberrant activation of the complement system in kidney diseases suggests its critical role in the long-term pathophysiology of renal damage of different etiologies (53). Evidence indicates the involvement of the complement system in aging-related diseases like Alzheimer’s, age-related macular degeneration, and osteoarthritis (81). Previously considered a protective mechanism against cancer, recent research identifies complement system and cellular senescence as main inducers of tumor growth in chronic, persistent inflammation contexts (e.g. in renal and prostate cancers) (82). Complement activation may also contribute to the pathogenesis of acute kidney injury, increasing the risk of subsequent progressive CKD, which may be mechanistically synonymous with accelerated ageing of the kidney (83).

A key mechanism in chronic inflammaging is cellular senescence, where cells become senescent through normal aging, telomere shortening, or DNA damage, hypoxia, oxidative or mitochondrial damage resulting in stress-induced premature senescence (84, 85). While in terminal growth arrest, senescent cells remain metabolically active and secrete factors contributing to chronic inflammation, renal fibrosis, and susceptibility of other cells to subsequent insults and senescence (62). ‘Immunosenescence of the adaptive immune system’ may contribute to uremic inflammation, resulting in systemic inflammation prevalent in advanced CKD (79). Disease-induced cellular senescence has been shown in kidneys and other organs and in patients with hypertension and type 2 diabetes (84).

The complement-inflammation axis causing premature inflammaging of the kidney involves several players:

▪ *Klotho expression*: anti-aging gene (complement down-regulates Klotho).

▪ *Pericyte/endothelial cell axis*: pericytes are a pivotal target of complement activation leading to a profibrotic maladaptive cellular response.

▪ *EndMT (endothelial-to-mesenchymal transition)*: Critical role of complement in induction of EndMT or prevention of EndMT by complement inhibition.

▪ *Pentraxin 3 (PTX3)*: main mediator of classical/lectin-mediated pathways of complement.

▪ *C1-Inhibitor*: prevents activation of all 3 complement pathways.

In HD patients, the risk of inflammaging is high due to multiple sources of inflammation causing immunological dysfunction and long-term complications affecting mortality (86). Immunological dysregulation, involving both the innate and adaptive response, plays a crucial role during HD sessions and chronic maintenance treatments (87). HD-induced inflammaging involves traditional and non-traditional risk factors which contribute to a persistent, systemic, pro-inflammatory, and pro-coagulant milieu, including conditions like diabetes, uremic toxins, genetic factors, or dialyzer biocompatibility (87). HD-induced inflammaging also contributes to the development and amplification of oxidative stress, cellular senescence, and persistent immune activation (complement system) (88). Vascular access and dialysis catheter contamination, as well as filter bioincompatibility, are exogenous risk factors dependent on material type and sterilization methods. Hemodialysis, as a model of extreme physiology in a vulnerable patient population, adds to the preexisting burden of the homeostatic/inflammatory milieu through recurrent complement activation upon contact with biomaterials during each treatment session (42, 43, 89).

### 3.3 Netosis: another piece of the bioincompatibility puzzle in hemodialysis

Since the early observation of complement and transient leukopenia during dialysis sessions, the complement-inflammation pathways triggered by HD procedures have been characterized in considerable detail. A recently described addition to the phenomenon of bio*in*compatibility are neutrophil extracellular traps (NETs), known to be involved in NETosis, a major harmful process in various pathophysiological conditions (90, 91).

Neutrophils play a crucial role in the first line of innate immune defence and produce NETs (extracellular fibers composed mainly of DNA from destroyed neutrophils) primarily to capture and kill bacteria and other pathogens, preventing their spread (92). Induction of NETosis results in neutrophil degranulation induced by reactive oxygen species (ROS), mainly from NADPH oxidase. NETs are beneficial due to their antimicrobial activity, but they also play a crucial role in the pathogenesis of diseases such as diabetes, cancer, thrombosis, lung disease, cardiovascular and autoimmune diseases associated with inflammatory conditions (90, 93). Proteomic analysis indicates that NETs induced by different stimuli are heterogeneous in terms of both protein composition and post-translational modifications, suggesting diverse biological effects under different conditions (94).

Uremia and the HD procedure are additional stimuli leading to NETs formation (95–97). Dysregulated neutrophil activities in the uremic milieu play a key role in vascular inflammatory responses, possibly caused by excessive NET formation which is associated with high mortality and CVD rates in ESKD (98).

The link between the dialysis procedure and membrane-induced NETosis establish another aspect of the complex bio*in*compatibility phenomena (97). Bieber et al. attributed neutrophil activation to extracorporeal components of the dialysis circuit and demonstrated that it occurs with each HD procedure, releasing NETs along with peroxidase activity, cfDNA, and calprotectin (99).

Considering bioincompatibility and NETs formation, it can be postulated that the uremic milieu with complement and inflammation marker uremic toxins, dialysis fluid contaminants like lipopolysaccharide (LPS), and the artificial surface of the membrane and the ECC collectively or individually provide stimuli for NETs generation in HD. These components may also be directly implicated in reactions leading to dialysis-induced systemic stress conditions with an adverse impact on multiple organs in dialysis patients. It is highly likely that NETs remain active during the interdialytic period, affecting systemic circulation and contributing to further end-organ damage, dialysis-induced systemic morbidity, and mortality. Further studies are needed to investigate NETosis kinetics during intradialytic and interdialytic periods to assess NET involvement in stress-targeted organs. NETs formation during HD and biological activity during the interdialytic period can be postulated as another integral facet of the bio*in*compatibility phenomenon in HD (88).

## 4 Mitigating risk and organ damage associated with bio*in*compatibility

In terms of bio*in*compatibility reactions in HD, the dialysis membrane stands out as the most potent stimulus for complement-inflammation responses, including cell senescence, inflammaging, and NETosis. A novel approach to improving overall biocompatibility involves surface modification of a polysulfone membrane with antioxidant Vitamin E stabilized polyvinylpyrrolidone contributing to creating a ‘pseudo-hydrolayer’ on the inner membrane surface (71, 100). This approach significantly reduces protein deposition, complement activation and platelet losses, as has been confirmed through clinical investigations that showed improved hemocompatibility profile markers of complement, cell and contact-coagulation activation (71). Together with advanced treatment modalities (e.g., high-volume OL-HDF) and a personalized HD approach (using treatment-guidance tools), such refinements in HD technology help minimize multiple pro-inflammatory insults encountered regularly by chronic HD patients (87, 101).

Recognizing the need for strategies to mitigate the detrimental consequences of bio*in*compatibility, progress has been made in terms of technological advancements and the mode of HD delivery (102–104) including artificial intelligence and machine learning support. Technology assistance’s added value lies in its contribution to the clinical decision process, ranging from identifying patients at risk to technology-directed treatment, leading to improvements in hard outcomes (105). Automated and self-adapting systems in smart dialysis machines, governed by adaptive algorithms with feedback control loops, offer innovative solutions (37, 106). An example is a sodium control module that has recently been validated in clinical trials (107–109) which contributes to cardioprotective hemodialysis through precise and personalized sodium and fluid management (4, 54). Artificial intelligence-supported systems enhance clinical assessment and management of key HD-related prescriptions with promising effects on outcomes (110–113).

## 5 Conclusions

In this narrative essay, we have explored the systemic consequences of bio*in*compatibility beyond traditional emphasis on membrane-related BMI phenomena. The complement-inflammation mediated manifestations of HD therapies extend well beyond procedure-related effects, impacting patient outcomes systemically over extended periods, not only in terms of the cardiovascular system but also impairing functions of various body organs (43). Accumulating evidence now suggests that processes of cellular senescence, inflammaging, and NETosis collectively cause dysfunction and long-term complications affecting mortality, constituting an integral component of the bio*in*compatibility equation in HD therapies (86). We provided future options including advances in polymer science and technical developments supported by artificial intelligence, to mitigate risk associated with bio*in*compatibility.

## Data Availability

The original contributions presented in the study are included in the article/supplementary material. Further inquiries can be directed to the corresponding author.
